# Preparation and Characterization of Oxide Nanotubes on Titanium Surface for Use in Controlled Drug Release Systems

**DOI:** 10.3390/ma17153753

**Published:** 2024-07-29

**Authors:** Patrycja Osak, Sandra Skwarek, Dariusz Łukowiec, Grzegorz Przeliorz, Bożena Łosiewicz

**Affiliations:** 1 Faculty of Science and Technology, Institute of Materials Engineering, University of Silesia in Katowice, 75 Pułku Piechoty 1A, 41-500 Chorzów, Poland; 2 Faculty of Mechanical Engineering, Silesian University of Technology, Konarskiego 18a, 44-100 Gliwice, Poland; 3 COMEF, Gdańska 2, 40-719 Katowice, Poland

**Keywords:** anodizing, corrosion resistance, drug delivery system, oxide nanotubes, titanium

## Abstract

Preventing or treating infections at implantation sites where the risk of bacterial contamination is high requires the development of intelligent drug delivery systems. The objective of this work was to develop a production method and characterization of fourth-generation oxide nanotubes on titanium grade 4 surface as a potential drug carrier. This study focused on the anodizing process; physico-chemical characterization using FE-SEM, EDS, and FTIR; in vitro corrosion resistance in an artificial saliva solution; and determining the drug release kinetics of gentamicin sulfate using UV-VIS. The anodizing process was optimized to produce fourth-generation oxide nanotubes in a fluoride-free electrolyte, ensuring rapid growth and lack of order. Results showed that the length of the oxide nanotubes was inversely proportional to the anodizing voltage, with longer nanotubes formed at lower voltages. The nanotubes were shown to have a honeycomb structure with silver particles co-deposited on the surface for antibacterial properties and were capable of carrying and releasing the antibiotic gentamicin sulfate in a controlled manner, following Fick’s first law of diffusion. The corrosion resistance study demonstrates that the oxide nanotubes enhance the corrosion resistance of the titanium surface. The oxide nanotubes show promise in enhancing osseointegration and reducing post-implantation complications.

## 1. Introduction

Titanium is characterized by biotolerance in the human body [1]. This metal also has low density, high corrosion resistance, and the ability to osseointegrate. Titanium is a vital element, which means that it does not have any harmful effects on the human body. Implants made of titanium are characterized by a long durability of 15–20 years. The constantly increasing number of toothless people and the desire to ensure the comfort of life have led to the more frequent implantation of dental implants made of titanium. However, the use of titanium implants does not fully meet the needs of modern medicine [1,2].

Surface treatment of dental implants increases the bioactivity of titanium implants [3,4]. Implantology also strives to use intelligent drug delivery systems (DDSs). Appropriate selection of surface treatment ensures the desired clinical effects. Surface treatment technologies are evolving very quickly. The anodized surface is best suited for use as drug carriers. This process is used in pharmacology, materials science, and biomedical engineering. In the anodizing process, a porous surface or one with oxide nanotubes can be obtained. Studies have shown that using the anodizing process does not leave harmful incompatible elements on the surface [3,4,5]. Additionally, anodizing is a simple, cheap, and easily scalable method that makes it possible to obtain oxide nanotubes with an ordered structure. Layers of oxide nanotubes on the titanium surface increase the substrate’s ability to mineralize bone tissue thanks to the developed surface and increased roughness.

Oxide nanotubes on titanium and its alloys are most often obtained in fluoride-based electrolytes [6]. Depending on the generation of the oxide nanotubes produced, by changing the current-time parameters and the amount of fluoride ions, nanotubes of various lengths and diameters can be obtained. Current trends aim to use reagents in the spirit of green chemistry, limiting the use of fluoride ions, which is why the fourth generation of oxide nanotubes is obtained in fluoride-free electrolytes. This type of electrochemical oxidation is characterized by the rapid growth of oxide nanotubes that are several microns long and lack order. The most commonly used electrolytes in the anodic production of fourth-generation oxide nanotubes are based on HCl [7,8], H_2_O_2_ [8,9], and their aqueous solutions, as well as mixtures of oxalic acid [10], formic acid [10], and sulfuric acid [11]. Fourth-generation oxide nanotubes are also obtained from aqueous solution of AgNO_3_ [12], SrNO_3_, NaNO_3_, and KNO_3_ [13]. The mechanism for obtaining oxide nanotubes from non-fluoride solutions is different than in the case of baths containing fluoride ions. After applying the appropriate voltage to the electrode, the anode current density increases rapidly and reaches its maximum values very quickly. The thickness of the obtained oxide reaches its maximum value immediately after the process starts, followed by an avalanche of breakdown and intensive oxygen evolution [12].

In the case of layers of oxide nanotubes obtained from nitrate baths, especially silver nitrate, along with obtaining a layer of oxide nanotubes, it is also possible to incorporate Ag particles inside the obtained nanotube structures during the anodizing process [12]. Silver is famous for its antibacterial properties, so it can be used in the treatment of peri-implantation infections, particularly *Staphylococcus aureus* [12,14,15], which is the most common cause of implant loss. Currently, the addition of silver nanoparticles is used as a bactericidal agent in medicine, as well as in disinfectants [16].

This work was undertaken to carry out innovative surface functionalization of titanium Garde 4 (Ti G4) by creating fourth-generation nanotubular oxide layers. The chosen method of modifying the Ti G4 surface by anodizing was aimed at shaping the surface morphology, structure, and properties for applications in intelligent DDSs. The first stage was the selection of the voltage, time, and type of electrolyte. In the second stage, the physico-chemical characterization of the obtained anode layers was carried out using field emission scanning electron microscopy (FE-SEM), energy dispersive spectroscopy (EDS), and Fourier transform infrared spectroscopy (FTiR). The third stage was to determine the mechanism and kinetics of the release of the drug substance in the form of gentamicin sulfate from the obtained oxide nanotubes based on UV-VIS analysis. GS is a broad-spectrum antibiotic, effective against a wide range of Gram-negative bacteria [17]. This makes it a valuable choice for preventing or treating infections at implant sites, where the risk of bacterial contamination is high. It is highly potent, meaning that even small amounts can be effective against bacteria. This is particularly beneficial when loading drugs into porous layers, as it allows for the effective use of the available space and material.

## 2. Materials and Methods

### 2.1. Substrate Preparation

The material used as an anodizing substrate was Ti G4 (Bibus Metals, Dąbrowa, Poland) in the form of a rod with a diameter of 10 mm and a length of 1500 mm after cold forming to increase mechanical properties and, in particular, strength. Ti G4 rods met the requirements of ISO 5832-2:2018-08 [18] and ASTM F67-13 [19]. The tested samples were prepared in the form of 5 mm-thick discs and then wet polished on silicon carbide abrasive papers of various gradations in increasing grit 600, 800, 1200, 3000, and 5000 using the metallographic grinding and polishing machine Metkon Forcipol 102 (Metkon Instruments Inc., Bursa, Turkey). After polishing, each sample was washed for 20 min in ultrapure water with a resistivity of 18.2 MΩ cm at a temperature of 25 °C (Milli-Q*^®^* Advantage A10 Water Purification System, Millipore SAS, Molsheim, France) using the USC 300 TH ultrasonic cleaner (VWR International, Radnor, PA, USA), ensuring effective removal of contaminants from elements with complex geometry. The washing procedure in ultrapure water was repeated twice.

### 2.2. Anodizing Conditions of Ti G4

For the anodizing process, electrodes were made from polished Ti G4 by attaching an insulated copper wire to the back of the titanium samples using silver glue to ensure electrical contact. The back side of the alloy samples and the side walls were protected with chemically resistant two-component epoxy resin. The electrodes were then cleaned with Milli-Q*^®^* water in an ultrasonic bath for 20 min and placed in the bath for the anodizing process in a two-electrode system. Anodizing was performed in an aqueous solution of 0.5% silver nitrate (Sigma-Aldrich, Saint Louis, MI, USA) at a thermostated temperature of 15(1) °C at a voltage from 50 to 80 V for 60 s. Anodizing was carried out using a high-current PWR800H power supply (Kikusui Electronics Corporation, Yokohama, Japan). The distance of the sample (anode), with a geometric area of 0.64 cm^2^ from the platinum mesh (cathode), with an area of 16 cm^2^, was constant and amounted to 20 mm. After the anodizing process, the electrodes were rinsed in Milli-Q*^®^* water and air-dried at ambient temperature.

### 2.3. FE-SEM Measurements

The surface morphology and thickness of the oxide nanotube layers on the Ti G4 surface were examined using a Hitachi HD-2300A field emission scanning electron microscope (FE-SEM) (Hitachi Ltd., Tokyo, Japan) under a low vacuum condition of 50 Pa at an accelerating voltage of 15 kV and using a Hitachi TM4000/TM4000Plus II microscope–Hitachi High Technologies (Hitachi Ltd., Tokyo, Japan) under low vacuum conditions at an accelerating voltage of 20 kV. All microscopic images were collected by secondary electrons (SE). The local chemical composition and surface distribution of elements were determined using an Energy Dispersive Spectrometer (EDS, AZtecLiveOne 30 mm^2^ Oxford Instruments, Abingdon, UK).

### 2.4. ATR-FTIR Measurements

Fourier attenuated total reflectance infrared spectroscopy (ATR-FTIR) was used to determine the functional groups of the obtained oxide nanotubes before and after antibiotic implementation. Radiation covering the infrared range from 4000 to 550 cm^−1^ was split into two beams, one of which followed a path of a fixed length, and the other was generated by an interferometer with a moving mirror moving at a constant speed. The changing difference in the path lengths of both beams caused mutual interference, resulting in an interferogram. The use of the Fourier transform allowed the interferogram to be transformed from the time domain to the frequency domain, i.e., spectrum. Transmittance measurements in the fundamental infrared range were based on the phenomenon of total internal reflection of light from the interface of two materials with different refractive indices. ATR-FTIR absorption spectra were recorded using a Shimadzu IR Prestige-21 FTIR spectrophotometer (Shimadzu, Kyoto, Japan) equipped with an ATR reflectance adapter with a diamond.

### 2.5. In Vitro Corrosion Resistance in Artificial Saliva Solution

The in vitro corrosion resistance test of the obtained electrodes was carried out in an artificial saliva solution (ASS) with pH = 7.4(1) at a temperature of 37(1) °C. The ASS consisted of NaCl—0.70 g dm^−3^, KCl—1.20 g dm^−3^, Na_2_HPO_4_—0.26 g dm^−3^, NaHCO_3_—1.50 g dm^−3^, and KSCN—0.33 g dm^−3^. A 4% NaOH solution and a 1% C_3_H_6_O_3_ solution were used to adjust the pH. Immediately before the measurements, a fresh portion of the solution was deaerated in 99.9999% pure argon (Air Liquide S.A., Paris, France) for 20 min.

All electrochemical studies were performed using a computer-controlled ModuLab XM ECS electrochemical system (Solartron Analytical, Farnborough, UK). Electrochemical measurements were carried out in a three-electrode system consisting of a working electrode (WE) in the form of the tested material, a counter electrode (CE) made of a platinum mesh, and a reference electrode (RE) placed in the Luggin capillary against which all potential values were measured. The reference electrode was a saturated calomel electrode (SCE) with a potential of 244.4(1) mV.

In vitro tests of the corrosion resistance of the obtained electrodes began with the measurement of the open circuit potential (E_OC_). According to ISO 10271:2021-02 [20], the measurement time for the E_OC_ change was 2 h with a sampling frequency of every 10 s. In E_OC_ measurements, the zero current galvanostatic potentiometry method with physical disconnection of CE was used. The obtained E_OC_ = f(t) curves were used for a preliminary assessment of the corrosion resistance of the tested electrodes. The stabilized E_OC_ value was considered as an approximate value of the corrosion potential (E_cor_) in further electrochemical tests.

The susceptibility to pitting corrosion was tested using anodic polarization curves in accordance with the ISO 10271:2022-02 standard [20]. The measurement was carried out from a potential of 150 mV more negative relative to the E_OC_ towards the anode potentials, recording the cathode–anode transition up to a potential of 4 V. Three measurement series were performed for each type of electrode, and the determined parameter values were given as average values with standard deviation (SD).

### 2.6. Gentamicin Sulfate Loading and Release from Oxide Nantubes on Ti G4

Fourth-generation oxide nanotube layers obtained on the Ti G4 surface were used as a drug carrier with controlled release of the drug substance. The drug release kinetics studies used an antibiotic in the form of gentamicin sulfate (GS) with the formula C_60_H_127_N_15_O_26_S (Merck KGaA, Darmstadt, Germany). 

The anodized Ti G4 samples were immersed in 7 mL of drug solution (50 mg mL^−1^). The initial drug dose was 350 mg for each sample. A Ti G4 sample with oxide nanotubes produced at a voltage from 50 to 60 V and a time of 60 s was immersed in the solution prepared in this way for 24 h. After 24 h, 1.5 mL of the solution containing GS was taken, and the amount of drug absorbed inside the oxide nanotubes was determined.

The amount of the absorbed drug was determined using the UV-VIS absorption spectroscopy method using electromagnetic radiation in the visible light (VIS) and near ultraviolet (UV) range. The work used a UV-Vis Biowave 2 (Biochrom WPA Biowave II UV/Visible Spectrophotometer, Cambridge, UK) spectrophotometer equipped with diode-array optics, enabling precise measurements with an open measurement chamber. The absorbance value was measured at a wavelength of λ = 245 nm, determining, in the first step, the absorbance value of the drug solution, and then the absorbance value of the collected solution after drug implementation.

The release kinetics of GS implemented inside the oxide nanotubes were examined by immersing the sample in 15 mL of phosphate buffer solution (PBS) at pH = 7.4(1) at 37 °C for 5 days. During the first hour, PBS was collected for analysis every hour, then every 24 h for 5 days. Each time, 1 mL of PBS was collected, and fresh solution was added. The amount of drug substance released from the oxide nanotubes was determined by UV-Vis spectroscopy. The absorbance was measured at a wavelength of λ = 250 nm in the first stage, determining the absorbance value of PBS and then the absorbance of the tested solution. The amount of GS released in percent by weight was determined from the drug calibration curve.

## 3. Results and Discussion

### 3.1. Formation of Oxide Nanotubes on Ti G4

**Figure 1:**
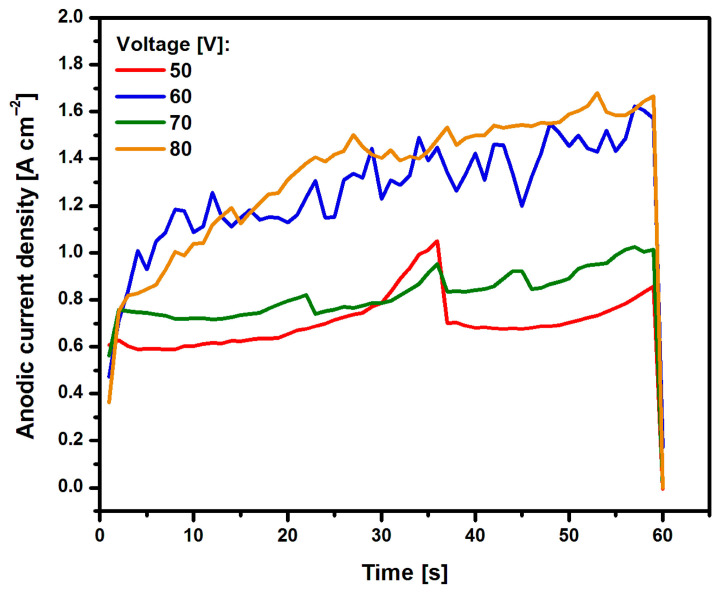
Dependence of current density (j) on time (t) for the Ti G4 electrode in 0.5% AgNO_3_ solution during anodizing at voltage from 50 to 80 V for 60 s.

The course of current densities as a function of time for Ti G4 electrodes anodized in a 0.5% AgNO_3_ solution is shown in Figure 1. It can be observed that as the voltage used in the anodizing process increased, the current density increased. The characteristic trough was not visible on the recorded curves j = f(t), resulting from the formation of oxide nanotubes of the first to third generation due to the presence of fluoride ions in the bath [21]. In the case of a fluoride-free bath, a sharp increase in current density was visible, as in Figure 1. This increase was caused by the fact that the emerging oxide film was produced as a result of the dissolution of oxides, which began at the electrolyte|oxide [22].

In the anodizing process in a fluoride-free bath, immediately after the start of electrochemical oxidation, the oxide layer is rapidly broken down, generating oxygen gas and a high current density value. Bauer et al. in [23] suggested that the oxide formed on the titanium surface has no resistivity, hence a high current can flow through the formed layer after reaching the breakdown voltage. During anodizing, the following electrochemical reactions occur in a fluoride-free solution [12]:

(1) $$\mathrm{Ti}\to {\mathrm{Ti}}^{4+}+4e^{-},$$

(2) $$2H_{2}O\to O_{2}↑+4H^{+}+4e^{-}.$$

**Figure 2:**
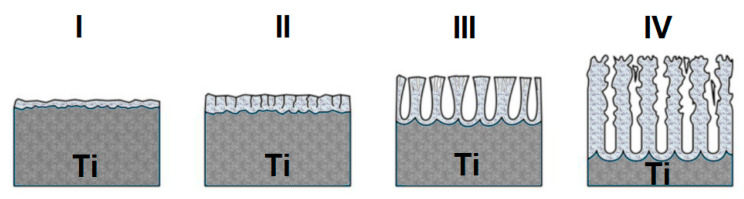
Scheme of anodic production of oxide nanotubes from a fluoride-free bath on the titanium surface: *(***I**)—barrier layer, (**II**)—increase in the thickness of the barrier layer and the formation of cracks, (**III**)—formation of a porous structure, (**IV**)—production of oxide nanotubes.

In the process of anodic production of oxide nanotubes, a number of reactions occur in parallel. Reactions mainly take place at the interface between the metal|oxide and metal|electrolyte phases [24,25,26]. Figure 2 shows a diagram of the formation of oxide nanotubes on the surface of titanium covered with a barrier layer, i.e., a self-passive TiO_2_ oxide layer. During the anodizing process, a constant voltage current flowed through the electrode. As a result of anodizing, the thin and self-passive barrier layer thickened (Figure 2I) [24]. With the time of the anodizing process, the electrical resistance of the system increased, and the thickness of the oxide increased, which cracked (Figure 2II) [24]. Then, the thick barrier layer further broke down, facilitating the penetration of the electrolyte inside, and a porous structure was formed (Figure 2III) [24]. Then, the stabilization and formation of nanotubes/nanopores on the surface of the material were observed (Figure 2IV) [24].

The anodizing process takes place in three stages [22,27,28]. During the electrochemical oxidation process, a very rapid release of bubbles from the anode surface is visible. In the anodizing process, an ionic current (j_i_) and an electronic current (j_e_) are generated [27]. J_i_ is responsible for the formation of oxide, and j_e_ is responsible for the formation of oxygen gas [12,27]. Oxide formation on the electrode surface influences the formation of the nanotube wall (Equation (1)), and oxygen evolution influences the nanotube to remain open (Equation (2)). As the anodizing voltage applied increases, the amount of released oxygen gas increases and, consequently, the current density increases. During the anodizing process, the thickness of the barrier layer increases, and when it approaches the critical thickness, a high current density value is generated, and the current value j_i_ decreases [22]. At the same time, a strong evolution of O_2_ gas begins at the boundary of the oxide film on the electrolyte side [28]. In the second stage, air bubbles accumulate on the electrode surface, creating the beginning of the formation of nanotubes. In the third stage, oxygen gas escapes from the interior of the oxide nanotubes, facilitating the electrolyte to reach the interior of the nanotubes. At the end of the production of nanotubes, the value of the current density decreases to a state close to zero [12,22,28].

### 3.2. FE-SEM/EDS Characterization of Oxide Nanutubes on TiG4

**Figure 3:**
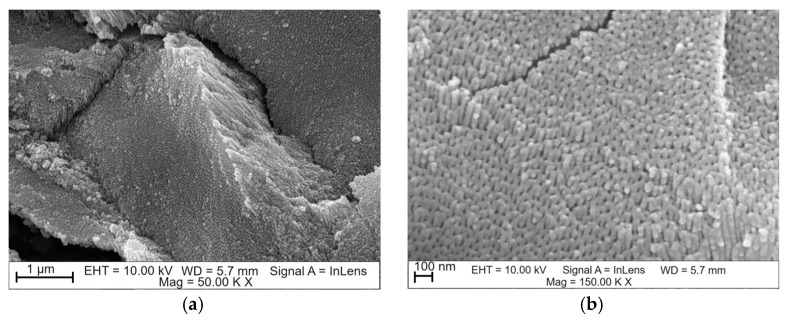

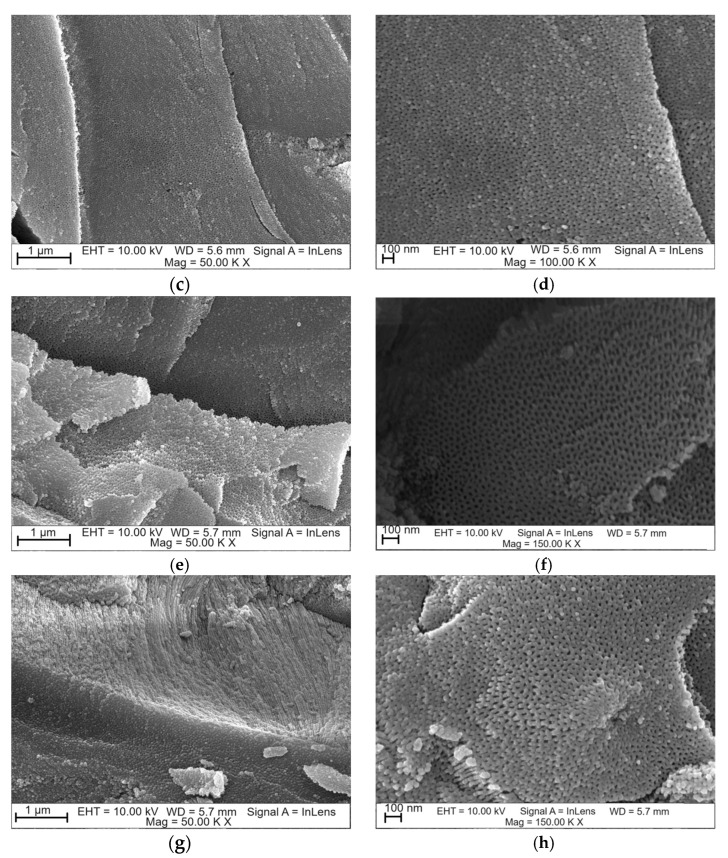
FE-SEM image of the microstructure of oxide nanotubes obtained on the Ti G4 surface by anodizing for 60 s at the voltage: (**a**,**b**) 50 V; (**c**,**d**) 60 V; (**e**,**f**) 70 V; (**g**,**h**) 80 V.

Based on FE-SEM images, the morphology and length of oxide nanotubes obtained on the Ti G4 surface under the proposed anodizing conditions were determined (Figure 3). The obtained microscopic images show an even distribution of single-wall oxide nanotubes. Each of the appropriately selected anodizing voltages ensured the production of fourth-generation oxide nanotubes.

Based on FE-SEM images, the length of oxide nanotubes was determined to be approximately 1.0(2) µm for the anodized surface at a voltage of 50 V, approximately 550(13) nm at a voltage of 60 V, approximately 375(11) nm at a voltage of 70 V, and approximately 230(8) nm at 80 V. On this basis, it can be concluded that as the anodizing voltage increased, the length of the obtained nanotubes decreased. After anodizing, each surface showed a honeycomb structure and multi-layer surfaces [29]. At a voltage of 50 V, nanopores/nanotubes formed, but their pores were clogged in some places with the remains of the oxide layer, which did not transform into nanotubes (Figure 3a,b). At a voltage of 60 V (Figure 3c,d) and 70 V (Figure 3e,f), no remnants of the unoxidized oxide film were observed on the surface of the oxide nanotubes. Figure 3g,h indicates that increasing the voltage to 80 V resulted in the formation of regular pores with a hexagonal structure [29,30]. In the literature, this type of structure has been obtained by two-stage anodic oxidation in acidic solutions [29,31,32]. In this work, honeycomb structures were obtained in a fast, one-step anodization process, lasting only 60 s. The hexagonal structure is used when nanotubes are used as carriers in intelligent drug delivery systems [30,32].

**Figure 4:**
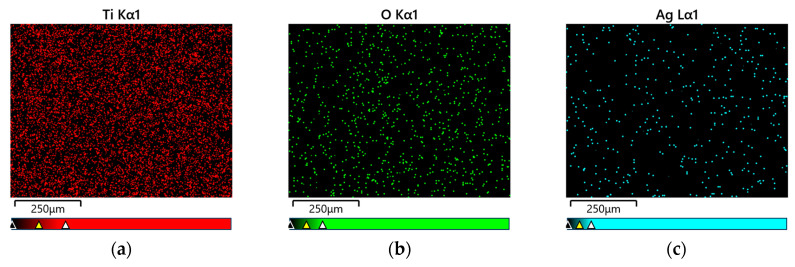
Map of the surface distribution of elements on the Ti G4 surface after anodizing in 0.5% AgNO_3_ solution at a voltage of 80 V: (**a**) Ti; (**b**) O; (**c**) Ag.

The use of AgNO_3_ solution in the Ti G4 anodizing process enabled not only the formation of layers of fourth-generation oxide nanotubes but also the co-deposition of silver particles. Figure 4 shows an example of the surface distribution of elements in the tested micro-area on the Ti G4 surface after the anodizing process at a voltage of 80 V. A uniform distribution of elements, such as titanium, oxygen, and silver in the examined micro-area on the surface of the obtained layer of oxide nanotubes, is visible in Figure 4.

**Figure 5:**
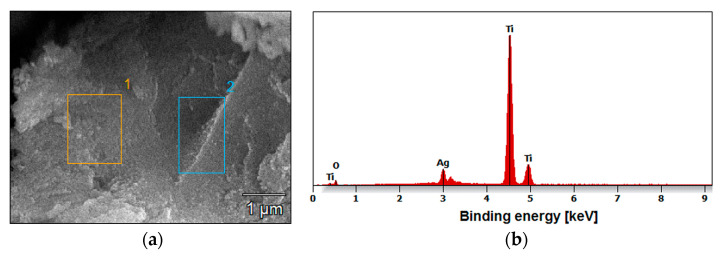
Micro-areas on the Ti G4 surface after anodizing in 0.5% AgNO_3_ solution at 50 V for 60 s: (**a**) FE-SEM image with the region 1 and 2 selected for EDS analysis; (**b**) EDS spectrum.

The amount of silver on the surface of all oxide nanotube layers obtained on a Ti G4 substrate was determined by EDS microanalysis. An example FE-SEM image of the tested micro-areas on the Ti G4 surface after anodizing in 0.5% AgNO_3_ solution at a voltage of 50 V and the recorded EDS spectrum are shown in Figure 5a,b, respectively. EDS analysis showed that by oxidizing Ti G4 at voltages of 50 and 60 V, approximately 1.4(2) wt.% Ag was incorporated into the anodized surface (Table 1). The increase in anodizing voltage increased the amount of silver in the oxide layer, which was 1.9(1) wt.% at 70 V and 2.2(5) wt.% at a voltage of 80 V (Table 1).

It should be noted that the presence of silver influences interactions with the bacterial structures of the biofilm that appears in the oral environment [33]. Using electrostatic forces, silver particles are attracted to the cytoplasmic membrane and the cell membrane wall. As a consequence, the membrane becomes semi-permeable and destroys bacterial structures [34]. The silver particles present in the oxide nanotube layer are small, so they have a positive effect on inhibiting bacteria after the implantation process. Too high a content of silver present on the surface may influence changes in the DNA structure and induce oxidative stress and genotoxicity [35].

Local measurement of the chemical composition of the tested materials using the microanalytical EDS method also showed the presence of titanium and oxygen. However, it should be emphasized that determining the percentage of oxygen using this method is difficult due to the fact that the characteristic radiation of light elements is absorbed more intensively by the sample. Moreover, atoms from ^5^B to ^10^Ne have only two shells filled with electrons, i.e., only one K emission series. The excitation energy of the K series is therefore very small and amounts to a maximum of 1 keV [36].

### 3.3. Assessment of In Vitro Corrosion Resistance in Artificial Saliva Solution

#### 3.3.1. Open Circuit Potential Study

**Figure 6:**
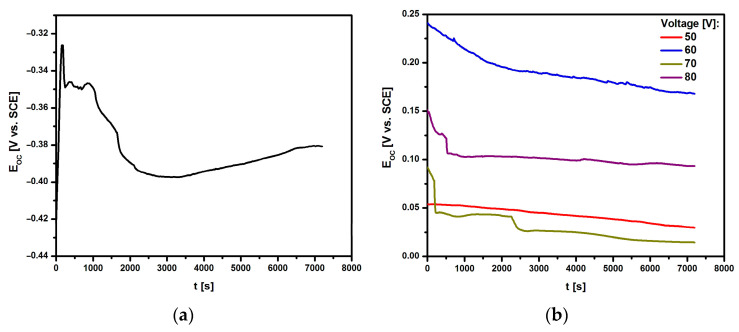
Dependence of the open circuit potential (E_OC_) on the immersion time (t) in artificial saliva solution at 37 °C for the Ti G4 electrode: (**a**) after mechanical polishing; (**b**) after anodizing at 50–80 V for 60 s in 0.5% silver nitrate solution.

Figure 6 shows the relationship of the open circuit potential on the immersion time in ASS at 37 °C for the Ti G4 electrode before and after anodizing at 50–80 V for 60 s in 0.5% silver nitrate solution. The obtained E_OC_ = f(t) curves were used for a preliminary assessment of the in vitro corrosion resistance of the tested electrodes.

For all tested electrodes, the E_OC_ value changed dynamically in the first 2000 s. Ion-electron equilibrium, manifested by a stable E_OC_ value, was achieved after 7200 s. The E_OC_ for the mechanically polished Ti G4 electrode was −0.381(4) V (Figure 6a). The use of surface modification by anodizing at voltages from 50 to 80 V shifted the E_OC_ value towards positive potentials at which the electrochemical oxidation process was more difficult (Figure 6b). The stable E_OC_ value was between 0.015(3) and 0.168(8) V depending on the anodizing voltage. Such a nature of the E_OC_ = f(t) curves indicated an increase in the corrosion resistance of Ti G4 as a result of the surface modification and improvement of the barrier properties of the oxide layer as a result of its thickening.

#### 3.3.2. Anodic Polarization Curves Study

**Figure 7:**
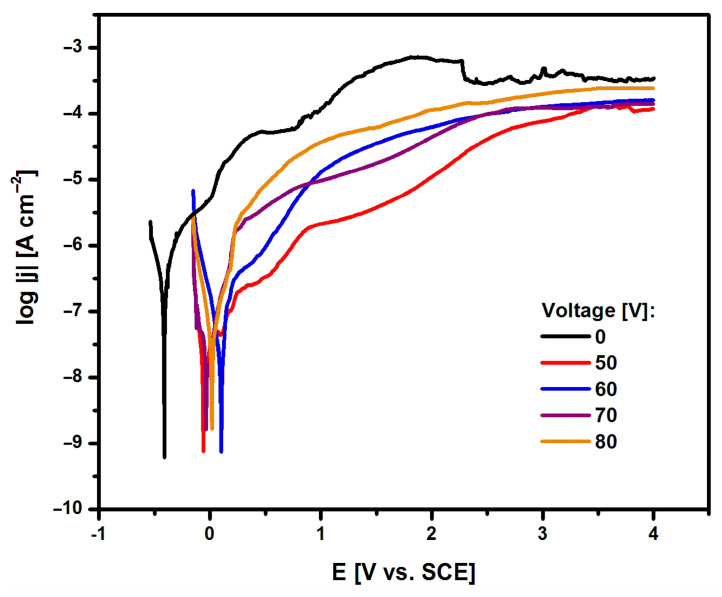
Anodic polarization curves for the Ti G4 electrode before and after anodizing at 50–80 V for 60 s in 0.5% silver nitrate solution, obtained in ASS at a temperature of 37 °C.

The susceptibility of the Ti G4 electrode to pitting corrosion in ASS containing aggressive chloride ions at a temperature of 37 °C was tested in the initial state, i.e., after mechanical polishing (0 V) and after anodizing at a voltage of 50–80 V for 60 s in 0.5% silver nitrate solution based on anodic polarization curves. During the measurement, the change in the potential of the tested electrodes was recorded using an electrode polarization rate of v = 1 mV s^−1^. The recorded anodic polarization curves are presented in the semi-logarithmic form log|j| = f(E) in Figure 7.

Based on the results obtained, it can be seen that the use of surface modification by creating layers of fourth-generation oxide nanotubes increased the corrosion resistance of Ti G4. Potentiodynamic characteristics for oxide nanotube layers obtained on the Ti G4 surface in the voltage range from 50 to 80 V for 60 s in a 0.5% AgNO_3_ solution showed a similar course. The lowest value of E_cor_ = −0.412(16) V, indicating the lowest corrosion resistance, was obtained for the Ti G4 surface before anodization with a self-passive oxide layer on the surface. In the case of the Ti G4 surface anodized at 50 V, the E_cor_ value was −0.059(7) V; at 60 V, the E_cor_ was equal to 0.099(10) V; at 70 V, the E_cor_ took a value of −0.035(9) V; and at 80 V, the E_cor_ was 0.018(8) V. The highest average j_cor_ value of approximately 3.39 × 10^−7^ A cm^−2^ was observed for the Ti G4 electrode after mechanical polishing, indicating the fastest kinetics of the corrosion process. The average j_cor_ value for the oxide nanotube layer obtained at 50 and 60 V was 7.59 × 10^−10^ A cm^−2^ and 7.36 × 10^−10^ A cm^−2^, respectively. For oxide nanotube layers obtained at higher voltages, i.e., 70 and 80 V, the j_cor_ value was 1.35 × 10^−9^ A cm^−2^ and 1.67 × 10^−9^ A cm^−2^, respectively. The tested Ti G4 electrodes with oxide layers on the surface showed corrosion resistance in the range of cathodic potentials and passive anodic behaviour. In the range of anodic potentials, electrochemical oxidation reactions occurred at values that are more positive compared to E_cor_. The obtained potentiodynamic characteristics confirm that the anodizing process of Ti G4 under the proposed electrochemical conditions increases the in vitro corrosion resistance of Ti G4, which can be used for long-term implants. Similar behaviour was observed in the literature for titanium and its alloys covered with oxide layers in biological environments [37,38,39,40].

### 3.4. Assessment of Oxide Nanotube Layers on Ti G4 as Drug Carriers

#### 3.4.1. ATR-FTIR Characterization of Anodized Ti G4 after GS Implementation

**Figure 8:**
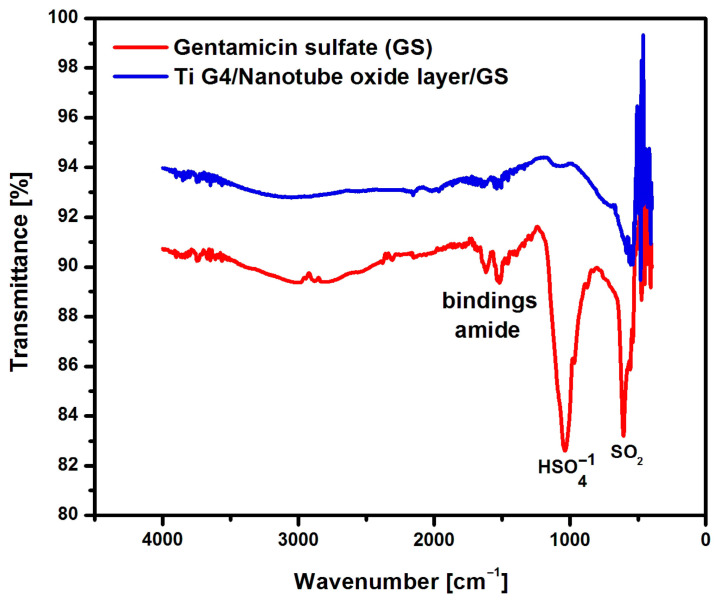
ATR-FTIR absorption spectrum obtained for gentamicin sulfate (GS) powder and Ti G4 sample with a layer of oxide nanotubes produced by anodization in 0.5% AgNO_3_ solution at 80 V for 60 s with implemented GS.

To assess the possibility of using the obtained layers of oxide nanotubes on a Ti G4 substrate as a potential drug carrier for applications in intelligent drug DDSs, gentamicin sulfate was implemented inside the nanotubes. ATR-FTIR characterization was performed on Ti G4 samples after anodization in 0.5% AgNO_3_ solution at 50, 60, 70, and 80 V for 60 s with absorbed drug and, comparatively, on gentamicin sulfate in the initial state in the form of powder (Figure 8). Based on the ATR-FTIR spectroscopic measurements, individual functional groups were assigned to specific areas with characteristic absorption bands.

Figure 8 shows an example ATR-FTIR absorption spectrum of gentamicin sulfate in the form of powder and the drug implemented inside the oxide nanotubes produced by anodization in 0.5% AgNO_3_ solution at 80 V for 60 s. The obtained ATR-FTIR absorption spectrum showed absorption bands with wave numbers 1616, 1558, and 1456 cm^−1^, which belonged to the type I amide bond and type II amide bond present in gentamicin sulfate [41]. The peak present at 1035 cm^−1^ was related to the ${\mathrm{HSO}}_{4}^{-1}$ group present in the drug structure. However, the peak visible at 607 cm^−1^ was caused by the SO_2_ band [42]. The ATR-FTIR absorption spectrum obtained for oxide nanotubes with the drug implemented confirmed the presence of gentamicin sulfate inside the nanotubes. In the oxide nanotubes loaded with gentamicin sulfate, small amide bands were visible in the range of 1616–1456 cm^−1^ and the spectrum at 607 cm^−1^, which proves the successful application of the drug into the interior of the fourth generation oxide nanotubes obtained on Ti G4 from an aqueous solution of 0.5% AgNO_3_.

#### 3.4.2. Release Kinetics of Gentamicin Sulfate from Oxide Nanotube Layers on Ti G4

Immediately after the implantation procedure, inflammation appears around the implants, which is the body’s natural reaction to a foreign body in the form of an implant. Gentamicin sulfate is an antibiotic with a broad biocidal effect that can be used to extinguish inflammation. This antibiotic inhibits bacterial protein synthesis by binding to the 30S ribosomal subunit. As a result of damage to the bacterial protein membrane, irreversible changes in mRNA occur, and bacterial growth is inhibited [41,43]. Gentamicin sulfate acts on Gram-negative bacteria such as *Klebsiella pneumoniae*, *Escherichia coli* and Gram-positive bacteria such as *Staphylococcus epidermidis* and *Staphylococcus aureus* [41]. The ability to load gentamicin sulfate into porous layers allows for localized delivery of the antibiotic directly to the site of the implant. This can help achieve high local concentrations of the drug without systemic side effects or the need for prolonged systemic antibiotic therapy. In order to determine the effect of the proposed anodization conditions, Ti G4 samples with an anodic layer of fourth-generation oxide nanotubes obtained at a voltage of 50 to 80 V for 60 s in 0.5% AgNO_3_ solution and implemented gentamicin sulfate were analysed for drug release kinetics.

Table 2 shows the amount of drug loaded inside the nanotubes depending on the voltage used in the anodic process. Based on FE-SEM images (Figure 3), it was determined that the length of the obtained nanotubes decreased as the anodizing voltage increased. The amount of gentamicin sulfate loaded correlated with the length of the oxide nanotubes. In total, 200(16) mg of the drug penetrated into the interior of the oxide nanotubes obtained at a voltage of 50 V, while for the shortest nanotubes obtained at a voltage of 80 V, 116(9) mg of gentamicin sulfate was loaded.

**Figure 9:**
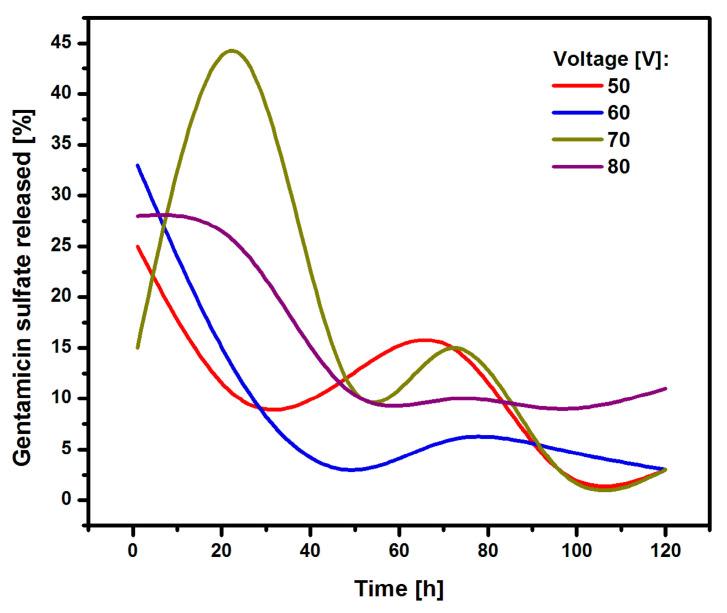
The amount of gentamicin sulfate released from oxide nanotube layers obtained by anodizing Ti G4 at a voltage from 50 to 80 V for 60 s in a 0.5% AgNO_3_ solution.

Figure 9 shows the amount of gentamicin sulfate released from the oxide nanotube layers on Ti G4 during immersion in the PBS solution for 120 h.

In the first hours, the drug was intensively released from the inside of the oxide nanotubes. In the case of the longest oxide nanotubes obtained at 50 V, approximately 25% of the drug was released in the first hour. In the case of nanotubes obtained at 60 and 80 V, the initial amount of drug released was approximately 25%. Oxide nanotubes obtained at 70 V showed a strong drug release from their interior of 45%. Nanotubes obtained at 50, 60, and 70 V showed a dynamic course in the subsequent hours of release, with an upward trend in drug release between hours 60 and 80 of the study. Nanotubes obtained at 80 V showed a constant therapeutic dose level from the 50th hour of release. This nature of drug release is related to the regular arrangement of the resulting nanotubes having a honeycomb structure. The obtained nanotubes were fully formed, which allowed the drug to penetrate evenly into the nanotubes. The gentamicin sulfate was released from the interior of the oxide nanotubes in accordance with Fick’s first law, which states that the amount of drug diffusing per unit time through the surface perpendicular to the direction is directly proportional to the area of this surface and the concentration gradient of the drug in the system [44].

Oxide nanotube layers offer a promising platform for drug delivery around implants due to their ability to provide controlled, localized, and potentially stimuli-responsive release of therapeutic agents. This technology has the potential to significantly improve the success rates of implants by reducing the risk of infection, promoting tissue healing, and enhancing patient comfort and compliance. The release of drugs from the interior of oxide nanotubes, in accordance with Fick’s first law, is a fundamental concept in the field of drug delivery and nanotechnology. Fick’s first law describes the diffusion process, which is a key mechanism for drug release from nanotubes and other controlled-release systems. According to literature data, oxide nanotubes are characterized by the presence of negatively charged electrostatic forces inside the nanotube [44,45]. Therefore, oxide nanotubes can be loaded with drug by physically trapping the drug inside the nanotube as well as by binding to positively charged gentamicin sulfate particles. A sample containing oxide nanotubes loaded with drug particles, immersed in the release solution, tends to equalize the concentration gradient of the drug and liquid inside the nanotube. This type of drug carrier is designed to operate in a biological environment where there is a constant gradient of liquid flow. The drug-loaded oxide nanotubes produced in this work were not covered with an additional polymer layer; therefore, the drug was released in accordance with Fick’s first law, because there were no processes of first absorption of fluids and, in the next stage, drug release.

## 4. Conclusions

Based on the results obtained, it can be concluded that one-step anodizing process in a 0.5% AgNO_3_ solution at 15(1) °C in the voltage range of 50–80 V for 60 s is an effective, scalable, and repeatable method for obtaining layers of fourth-generation oxide nanotubes on Ti G4, which does not leave harmful elements on the surface. The FE-SEM study showed that the obtained oxide nanotubes had a length of 1.02(2) µm at an anodizing voltage of 50 V, 550(13) nm at 60 V, 375(11) nm at 70 V, and 230(8) nm at 80 V. An increase in anodizing voltage reduced the length of the obtained oxide nanotubes but affected the formation of regular hexagonal nanotubes that form a honeycomb structure. During the anodizing process, silver particles up to 2.2(5) wt.% were co-deposited on the surface of Ti G4 for antibacterial properties, the presence of which was confirmed by EDS analysis. Based on electrochemical tests using the open circuit potential method and anodic polarization curves, it was found that the obtained oxide nanotube layers showed higher corrosion resistance compared to the Ti G4 surface not modified in ASS as. Assessment of the anodized Ti G4 as gentamicin sulfate carrier carried out using ATR-FTIR and UV-VIS absorption spectroscopy revealed that the obtained fourth-generation oxide nanotubes can be a universal drug carrier in intelligent targeted drug delivery systems. The antibiotic in the form of gentamicin sulfate loaded into the obtained oxide nanotubes was released in accordance with Fick’s first law. The release kinetics were influenced by the length and structure of the nanotubes. Gentamicin sulfate was dynamically released from the oxide nanotube layers obtained at 50 and 60 V, but the most effective therapeutic dose could be obtained for nanotubes obtained at 70 and 80 V. Fourth-generation oxide nanotube layers obtained under selected anodizing conditions can serve as drug carriers, which will affect to accelerate osseointegration processes and reduce post-implantation complications.

## Figures and Tables

**Table 1:** Quantitative analysis of the elements present on the surface of oxide nanotube layers obtained on a Ti G4 substrate in a 0.5% AgNO_3_ solution.

Voltage (V)	Element (wt.%)		
	O	Ti	Ag
50	35.8(2)	62.8(4)	1.4(2)
60	38.1(1)	61.5(2)	1.4(1)
70	25.0(7)	73.0(3)	1.9(1)
80	30.9(2)	66.9(1)	2.2(5)

**Table 2:** The amount of gentamicin sulfate implemented inside the fourth-generation oxide nanotubes on the Ti G4 surface obtained at a voltage from 50 to 80 V for 60 s in 0.5% AgNO_3_ solution.

Voltage (V)	Amount of Gentamicin Sulfate Loaded (mg)
50	200(16)
60	175(14)
70	147(12)
80	116(9)

## Data Availability

Data are contained within the article.
